# Pregnancy in Pompe disease

**DOI:** 10.1186/1471-2474-14-S2-O11

**Published:** 2013-05-29

**Authors:** Ursula Plöckinger

**Affiliations:** 1 Interdisciplinary Centre of Metabolism: Endocrinology, Diabetes and Metabolism, Charité Universitätsmedizin Berlin, Berlin, Germany

## 

Pregnancy in Pompe disease is still a rare event. Only few reports have been published. Physiological changes of pregnancy may be aggravated by Pompe disease or vice versa. Both may pose a risk for the mother and the unborn child. This talk outlines physiological changes of the cardiovascular, respiratory and hormonal system during pregnancy and delineates the impact on a 36-year-old Pompe patient with a twin pregnancy. Furthermore, recommendations for a prospective medical strategy are presented and a workflow for routine follow-up during pregnancy is suggested. Recommendations include the planning of the pregnancy, genetic counselling, and pre-pregnancy investigations. An interdisciplinary team consisting of an obstetrician, pulmonologist, internist, endocrinologist and anaesthesiologist is suggested for care of the pregnant patient with Pompe disease.

